# Examination of Curcumin and Fenugreek Soluble Fiber Supplementation on Submaximal and Maximal Aerobic Performance Indices

**DOI:** 10.3390/jfmk5020034

**Published:** 2020-05-30

**Authors:** Jensen Goh, Walter Menke, Lauren P. Herrick, Marilyn S. Campbell, Mark G. Abel, Bradley S. Fleenor, Haley C. Bergstrom

**Affiliations:** 1 Department of Kinesiology and Health Promotion, University of Kentucky, Lexington, KY 40506, USA; jensen.goh@uky.edu (J.G.); walter.menke@uky.edu (W.M.); lauren.herrick3@gmail.com (L.P.H.); mca243@uky.edu (M.S.C.); mgabel2@uky.edu (M.G.A.); 2 School of Kinesiology, Ball State University, Muncie, IN 47306, USA; bsfleenor@bsu.edu

**Keywords:** curcumin, galactomannan, ventilatory threshold, nutritional intervention, performance

## Abstract

This study examined the effects of curcumin and fenugreek soluble fiber supplementation on the ventilatory threshold (VT) and peak oxygen consumption ($\dot{V}$O_2_ peak). Methods: Forty-five untrained men and women were randomly assigned to one of three supplementation groups: placebo (PLA, *n* = 13), 500 mg·day^−1^ CurQfen^®^ (CUR, *n* = 14), or 300 mg·day^−1^ fenugreek soluble fiber (FEN, *n* = 18). Participants completed a maximal graded exercise test on a cycle ergometer to determine the VT and $\dot{V}$O_2_ peak before (PRE) and after (POST) 28 days of daily supplementation. Separate, one-way analyses of covariance (ANCOVAs) were used to examine the between-group differences for adjusted POST VT and $\dot{V}$O_2_ peak values, covaried for the respective PRE-test values. Results: The adjusted POST VT $\dot{V}$O_2_ values for the CUR (mean ± SD = 1.593 ± 0.157 L·min^−1^) and FEN (1.597 ± 0.157 L·min^−1^) groups were greater than (*p* = 0.039 and *p* = 0.025, respectively) the PLA (1.465 ± 0.155 L·min^−1^) group, but the FEN and CUR groups were not different (*p* = 0.943). There were no differences in the adjusted $\dot{V}$O_2_ peak values (F = 0.613, *p* = 0.547) among groups. Conclusion: These findings indicated that fenugreek soluble fiber was responsible for the improvements in the submaximal performance index for both CUR and FEN groups.

## 1. Introduction

Curcumin is a polyphenol that targets multiple signaling pathways and has been shown to positively influence health at the cellular level [1]. It is an active ingredient of a rhizomatous herbaceous perennial plant of the ginger family called turmeric and has been widely used as a spice and medicine in various cultures throughout history [1,2]. These uses range from colorants, cosmetics, teas, and taste enhancers to anti-inflammatory agents and supplements. In populations where curcumin (100–200 mg·day^−1^) is consumed, epidemiological data have indicated the incidences of some chronic diseases (e.g., large bowel cancer) are lower, compared with populations of non-consumption [3,4]. Curcumin has been shown to have strong antioxidant, anti-hypertensive, anti-inflammatory, and anti-diabetic affects as well as potential body composition benefits and positive mediation of various cardio-health risk markers [5,6,7]. There is evidence from both murine models and human studies that curcumin supplementation improved the vascular restructuring and endothelial dysfunction prevalent in diabetes, metabolic syndrome, and hypertension [2,7]. In addition, curcumin supplementation has been shown to upregulate the production of endogenous nitric oxide (NO) production [1,2,7], which mediates endothelial-dependent vasodilation. It is possible that curcumin may enhance blood flow to the working cardiac and skeletal muscles and positively influence aerobic exercise performance.

One of the primary limitations to curcumin supplementation is its poor bioavailability. Curcumin has poor absorption, rapid metabolism, and rapid systemic elimination [6,8,9]. These characteristics result in the tendency for curcumin supplementation alone to not effectively increase plasma and tissue concentrations of curcumin to physiologically relevant values of 0.1 micromolar [10]. It has been documented [6] that, even at high doses (12 g·day^−1^), the plasma and tissue concentrations of curcumin may still be lower than the necessary threshold for physiological effects under short-term supplementation periods (<6 weeks). Therefore, approaches to slow digestion, increase absorption, and reduce systemic elimination of curcumin have been examined [9,11,12]. For example, curcumin has been combined with piperine, which interferes with liver glucuronidation [12], or fenugreek soluble fiber, which slows the release and protects curcumin from acidic gastrointestinal conditions [8]. Curcumin, combined with fenugreek soluble fiber (galactomannans), has been shown to enhance curcumin bioavailability by increasing the absorption and saturation to up to 20 times compared to curcumin alone [8,9].

The main role of fenugreek soluble fiber in combination with curcumin is to increase plasma and tissue concentrations of curcumin by slowing down its digestion and elimination [8,13,14]. However, it is important to note that the galactomannan component of fenugreek soluble fiber has potential physiological effects [14,15,16]. Previous investigators have shown significantly slower gastric emptying, increased plasma sensitivity, decreased plasma insulin levels, reduced hepatic cholesterol concentration, and enhanced plasma free fatty acid (FFA) levels in circulation after 28 days of fenugreek galactomannan supplementation [14,15]. These effects, particularly decreased plasma insulin levels and increased FFA levels, have been linked to increased rates of FFA oxidation [13,14,17]. Thus, in addition to increasing the absorption of curcumin from the small intestine, fenugreek may also have the potential to improve metabolic parameters associated with aerobic exercise performance.

Several previous investigators have examined the effects of curcumin in high relative doses (100 mg·kg^−1^ in mice), or in combination with bioavailability enhancing ingredients, on indices of vascular function and other markers of cardiovascular health [1,2,18]. The purported effects of curcumin in various forms on inflammatory pathways, nitric oxide production, and as an anti-oxidant [2,5,19], have recently lead to the examination of its potential as an ergogenic aid to delay fatigue and enhance recovery from exercise [19,20,21]. Orally optimized and high relative doses of curcumin supplementation have been shown to significantly decrease cytokine production in inflammatory pathways in mice as well as reduce various markers of exercise-induced muscular damage (EIMD) from repeated eccentric muscle actions in humans [20,21]. These effects have resulted in lower decrements in grip strength after fatiguing eccentric exercise [19] compared with placebos. There is also evidence that curcumin (at varying doses) may increase glycogen stores following 28 consecutive days of supplementation [19] and decrease the accumulation of metabolic byproducts (i.e., hydrogen ions, ammonia, etc.) [5], which may increase the time to fatigue and enhance recovery from long periods (>60 min) of exercise [19,20,21]. For example, Huang et al. (2015) showed curcumin supplementation significantly increased swim time to exhaustion in mice, dose-dependently, while decreasing injury markers by approximately fifty percent, when compared to the placebo. Thus, currently, there is evidence that curcumin in relatively high doses, or in combination with bioavailability boosters, may enhance endurance performance and increase time to exhaustion as well as improve recovery from EIMD [19,20,21].

Fatigue thresholds such as the ventilatory threshold (VT) provide a non-invasive assessment of metabolic responses during incremental exercise [22,23]. Theoretically, the VT demarcates the moderate to heavy exercise intensity domain [24,25] and provides information about the exercise intensity above which aerobic adenosine triphosphate (ATP) production is supplemented with anaerobic energy metabolism. Exercise performed above the VT (within the heavy domain) results in increased blood lactate concentration and hydrogen ion (H^+^) production [25]. The VT reflects the increased ventilation ($\dot{V}$_E_), relative to oxygen consumption ($\dot{V}$O_2_) in response to excess carbon dioxide (CO_2_) generated from the bicarbonate buffering of the H^+^ [22]. The VT has been used to assess physical fitness in both clinical [26] and athletic populations [27] and has been shown to be sensitive to training and nutritional intervention [28].

Previous studies have indicated the potential for curcumin supplementation to increase NO production, decrease metabolic byproduct accumulation [5], and increase time to exhaustion (T_lim_) [19,20,21]. It is possible these effects may also improve submaximal (VT) and maximal ($\dot{V}$O_2_ peak) indices of aerobic endurance performance. In addition, there is evidence that the galactomannan component of fenugreek soluble fiber, used to enhance the bioavailability of curcumin, may also have potential effects on upregulating FFAs [13,14]. This may delay the reliance on anaerobic energy production and increase the VT. No previous studies, however, have examined the effects of curcumin in combination with fenugreek soluble fiber and/or fenugreek soluble fiber alone on submaximal and maximal endurance performance markers such as the VT and $\dot{V}$O_2_ peak. Therefore, the purpose of this study was to examine the effects of curcumin + fenugreek soluble fiber and fenugreek soluble fiber supplementation on the VT and $\dot{V}$O_2_ peak. We hypothesized that 28 days of curcumin + fenugreek and fenugreek soluble fiber supplementation would result in increases in the VT and $\dot{V}$O_2_ peak compared to a placebo.

## 2. Materials and Methods

### 2.1. Experimental Approach

This study used a randomized, double-blind, placebo-controlled, parallel design with two experimental groups and one placebo group. Forty-five participants were randomly assigned to the placebo group (PLA, *n* = 13), curcumin + fenugreek supplement, CurQfen^®^ (CUR, *n* = 14), or fenugreek soluble fiber supplement (FEN, *n* = 18). The participants visited the testing center a total of six times; the second and sixth sessions lasted approximately two hours, and there were weekly check-in visits (four in total = visits three through six) during the 28-day supplementation period. During the first visit, each subject completed a health history questionnaire and signed an informed consent document. During the second visit, the participants completed a PRE-test graded exercise test (GXT_pre_ prior to 28 days of supplementation) to determine the PRE-test VT and $\dot{V}$O_2_ peak, followed by a 28-day supplementation protocol. The participants were asked to ingest one dose in capsule form (PLA, CUR, or FEN) every day for 28 days and one dose 60 min prior to the POST-test (GXT_post_ after 28 days of supplementation). The GXT was used to determine the PRE-test VT and $\dot{V}$O_2_ peak. Following 28-days of supplementation, each subject completed a POST-test GXT to determine the POST-test VT and $\dot{V}$O_2_ peak. Dietary intakes three days prior to the PRE- and POST-test days were recorded with food logs. In addition, supplement compliance was recorded with dosing logs. The primary outcomes were the VT and $\dot{V}$O_2_ peak and a secondary outcome was the respiratory exchange ratio (RER) at the VT. The total kilocalories and grams of each macronutrient determined from the dietary analyses were measured for descriptive purposes.

### 2.2. Participants

**Figure 1:**
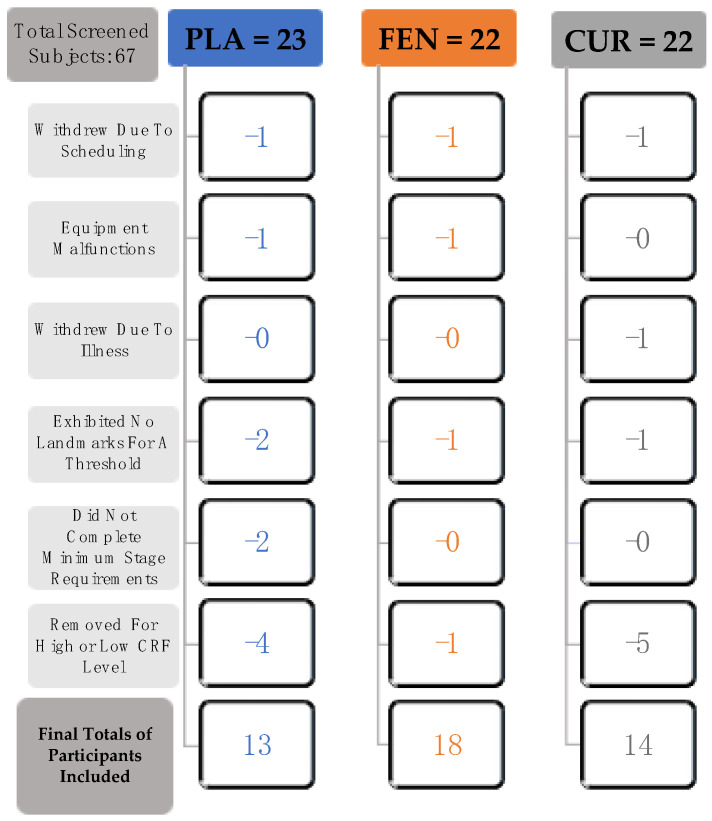
Flow chart demonstrating the process for inclusion of participants in the placebo (PLA, *n* = 13), fenugreek soluble fiber (FEN, *n* = 18), and curcumin+fenugreek soluble fiber (CUR, *n* = 14) groups. See participants section within the Methods for further descriptions of each of the criteria indicated in the flow chart. Note: cardiorespiratory fitness (CRF).

The sample size was determined from the sample size previously reported in a study (35) that examined the effects of a nutritional supplement intervention of the same dependent variables used in this study. In total, 67 participants were screened and enrolled in the study (Figure 1). Three of the participants withdrew due to scheduling conflicts, two of the participants were excluded due to equipment malfunctions, and one subject was excluded due to inability to complete PRE/POST-test measures as a result of illness. Four of the participants were excluded because they did not exhibit landmarks for threshold calculation, and two were excluded due to inability to complete minimal stage requirements needed for this test. The participants were untrained in aerobic exercise and engaged in no more than four hours of recreational activity per week. To account for variations in low- and high- fitness levels, participants were excluded if they fell below (very poor) or above (superior) the 10th percentile of cardiorespiratory fitness based on age and sex, according to the American College of Sports Medicine [29]. Five of the participants had a $\dot{V}$O_2_ peak that was below, and five of the participants had a $\dot{V}$O_2_ peak that was above the 10th percentile for cardiorespiratory fitness and were excluded from the analyses. Thus, 45 men (*n* = 24) and women (*n* = 21) (age: 21.2 ± 2.4 years; height: 174.4 ± 8.2 cm; body mass: 73.1 ± 13.4 kg; and body mass index (BMI): 24.0 ± 3.5 kg⋅m^−2^) completed this study (PLA = 13, FEN = 18, CUR = 14). All of the participants completed a health history questionnaire and met the following criteria: (a) no history of medical or surgical events that could significantly affect experimental results or increase the participants risk of injury, including cardiovascular, metabolic, renal, or hepatic disease as well as musculoskeletal disorders; (b) were not taking any medication that could significantly affect experimental results (e.g., vasodilators/vasoconstrictors); (c) were not currently using any nutritional supplements that could significantly affect experimental results; and (d) were not presently participating in another clinical trial or consuming another investigational product. The participants were instructed to not consume any caffeine on the testing day and avoid alcohol consumption for 24 h prior to testing. The study was approved by the Institutional Review Board for Human Participants at the University of Kentucky (IRB # 45965), and all participants signed a written informed consent document before testing.

### 2.3. Supplementation

A limitation to curcumin supplementation is its low bioavailability. The supplement CurQfen^®^ combines curcumin and fenugreek soluble fiber to significantly increase plasma concentrations of curcumin [8]. Galactomannan soluble dietary fiber from fenugreek seeds slows the digestion and rapid elimination of curcumin to allow better absorption into the bloodstream, improving absorption by 15.8 times of the curcumin standalone [8,9]. The 500-mg CurQfen^®^ capsule (Akay Group, Ltd., Dubai, UAE) contained 190 mg of total curcuminoids (curcumin—81%, demethoxycurcumin—15.7% and bisdemethoxycurcumin—2.6%). The 300-mg FenuMAT capsule (Akay Group, Ltd., Dubai, UAE) contained de-bitterized fenugreek dietary fiber, containing 75% to 80% galactomannans with 2–4% moisture. The fenugreek soluble fiber only group was included to account for any extraneous effects of fenugreek soluble fiber and the capsule contained 300 mg. The placebo capsule contained only microcrystalline cellulose. To maintain the double-blind nature of the study, the participants received the capsules in an opaque bottle and were not made aware of the appearance of the capsules of the other conditions. The participants consumed one dose daily and received the capsules on a weekly basis according to their randomly assigned group of PLA, CUR, or FEN. The capsules were ingested with 16 oz. of water every morning before eating for 28 days. The participants completed a dosing log and checked in weekly with their supplement bottles to ensure adherence to proper dosing procedures and to receive the following week’s supplements. The dosing log was used to check compliance (compliance = (# of doses taken/total # of doses provided) × 100). A compliance rate of >80% was required for inclusion in the data analyses. In addition, during supplementation, the participants were instructed to keep a three-day food log prior to each testing session and were asked not to change their diet and activity level during the study. The three-day food logs prior to each testing day were further analyzed to ensure dietary consistency. A total of 38 of the 45 participants (PLA = 11, FEN = 15, CUR = 12) completed and returned food logs that were used for subsequent analyses.

### 2.4. Graded Exercise Test

Each subject performed an incremental cycle test to exhaustion on an electronically braked cycle ergometer (Lode Corival, Groningen, The Netherlands) to determine the VT and $\dot{V}$O_2_ peak. The participants were familiarized with the equipment before proceeding with the GXT. The ergometer seat height was adjusted so that the subject’s legs reach near full extension at the bottom of the pedal revolution. Toe clips were used to maintain pedal contact throughout the test and all participants were equipped with a nose clip and a two-way valve mouthpiece to collect all expired air. A calibrated metabolic cart (TrueMax 2400, ParvoMedics, Sandy, UT, USA) was used to collect and analyze the expired gas samples. The gas analyzers were calibrated with room air and gases of known concentration prior to all testing sessions. The O_2_, CO_2_, and ventilatory parameters were expressed as 30 s averages. In addition, the heart rate was recorded with a Polar Heart Rate Monitor (Polar Electro Inc., Lake Success, NY, USA) that was synchronized with the metabolic cart. The Borg Rating 6–20 of Perceived Exertion (RPE) scale was used to quantify the subjective effort of the participant at the end of each minute during the test [30]. Following a one-minute warm up at 0 W, the resistance was increased to 50 W, and was then increased by 30 W every 2 min until the participants were unable to maintain 70 rev·min^−1^, or until volitional fatigue. This protocol was consistent with the study protocol previously used to assess $\dot{V}$O_2_ peak, gas exchange and ventilatory thresholds, as well as the electromyographic fatigue threshold in college-aged males [31]. The $\dot{V}$O_2_ peak was defined as the highest $\dot{V}$O_2_ value in the last 30 s of the test that met two of the following three criteria: (1) ≥ 90% of age-predicted heart rate; (2) respiratory exchange ratio (RER) ≥ 1.1; and (3) a plateau in oxygen uptake (less than 150 mL·min^−1^ in $\dot{V}$O_2_ over the last 30 s of the test).

### 2.5. Determination of the Ventilatory Threshold

**Figure 2:**
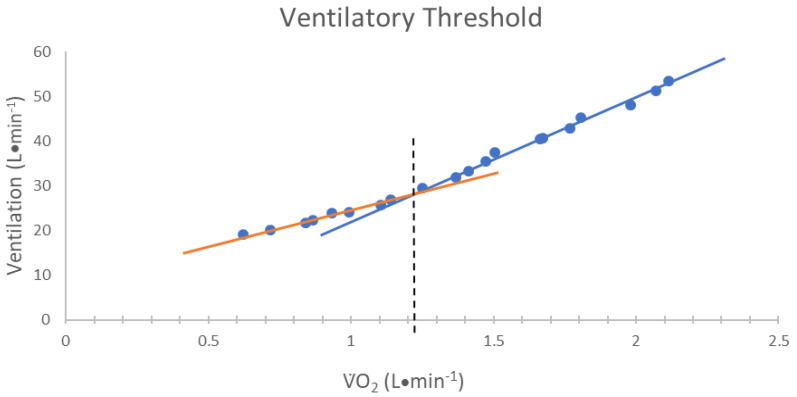
The method used for determining the ventilatory threshold (VT). The VT was defined as the oxygen consumption ($\dot{V}$O_2_) value corresponding to the intersection of two linear regression lines derived separately from data points below (orange line) and above (blue line) the breakpoint in the minute ventilation ($\dot{V}$_E_) relative to $\dot{V}$O_2_ relationship [22].

The VT was determined by using the V-slope method [22]. Specifically, the VT was determined from the $\dot{V}$_E_ versus $\dot{V}$O_2_ relationship. The VT was defined as the $\dot{V}$O_2_ value that corresponded with the point of non-linear increase in $\dot{V}$_E_ relative to $\dot{V}$O [22] (Figure 2). In addition, the RER at the VT was recorded.

### 2.6. Statistical Analyses

Separate, one-way analyses of variance (ANOVA) were used to determine if there were any significant differences among the PLA, FEN, and CUR groups for age, height, body mass, ventilatory threshold (VT$\dot{V}$O_2_), or $\dot{V}$O_2_ peak prior to supplementation. The PRE- and POST-test values for the PLA group (*n* = 13) were used for the calculation of test-retest reliability of the VT$\dot{V}$O_2_ and $\dot{V}$O_2_ peak, which consisted of the intraclass correlation coefficient model 2,1 (ICC_2,1_), the standard error of the measurement (SEM), and the minimal difference needed to be considered real (MD) for each dependent variable (VT$\dot{V}$O_2_ and $\dot{V}$O_2_ peak) [32]. The SEM was calculated as the SD × $\sqrt{1-ICC}$ and the MD was calculated as the SEM × 1.96 × $\sqrt{2}$ [32]. In addition, three separate paired sample t-test were used to determine if there were any significant changes in the dependent variable for the PLA group from PRE- to POST-test. Separate 2 (Time: PRE and POST) × 3 (Group: PL, CUR, FEN) mixed factorial ANOVAs were performed for the total kilocalories and grams for each macronutrient (carbohydrates, fats, and proteins) as well as for body mass and BMI. Three separate, one-way analyses of covariance (ANCOVA) (VT, RER at the VT, and $\dot{V}$O_2_ peak) were used to determine if there were any differences between adjusted POST-test values (VT$\dot{V}$O_2_, RER at the VT, and $\dot{V}$O_2_ peak), covaried for the respective PRE-test values [33]. Post-hoc analyses consisted of independent samples *t*-tests. Measures of effect size (Partial eta squared ($\eta_{p}^{2}$) and Cohen’s *d*) were calculated for al ANOVAs and paired sample *t*-tests, respectively. (The analyses were conducted using the Statistical Package for the Social Sciences software (v. 24.0 IMB SPSS Inc., Chicago, IL, USA). An alpha level of *p* ≤ 0.05 was considered statistically significant for all analyses.

## 3. Results

### 3.1. PRE- and POST- Test Descriptive Statistics, Supplement Compliance, and Dietary Recall

The data are presented as mean (SD) with 95% confidence intervals (CI), unless otherwise noted. The results of the one-way ANOVAs comparing PRE-test values indicated that there were no significant mean group differences for the VT$\dot{V}$O_2_ (PLA = 1.507 ± 0.325, FEN = 1.480 ± 0.328, CUR = 1.514 ± 0.440 L⋅min^−1^; F = 0.039, *p* = 0.961, $\eta_{p}^{2}$ = 0.002) or $\dot{V}$O_2_ peak (PLA = 39.06 ± 6.12, FEN = 37.70 ± 5.23, CUR = 40.214 ± 4.91 mL⋅kg^−1^⋅min^−1^; F = 0.068 *p* = 0.934, $\eta_{p\;}^{2}$ = 0.003) determined from the GXT, as well as for age (F = 1.753, *p* = 0.186, $\eta_{p}^{2}$ = 0.077), height (F = 0.241, *p* = 0.787, $\eta_{p}^{2}$ = 0.011), or body mass (F = 1.001, *p* = 0.376, $\eta_{p}^{2}$ = 0.046) values (Table 1). The POST-test values for the PLA, FEN, and CUR groups for the VT$\dot{V}$O_2_ were 1.473 ± 0.372, 1.579 ± 0.371, and 1.608 ± 0.426 L⋅min^−1^, respectively. The POST-test values for the PLA, FEN, and CUR groups for $\dot{V}$O_2_ peak were 39.57 ± 7.56, 37.50 ± 6.08, 40.59 ± 5.78 mL⋅kg^−1^⋅min^−1^, respectively. There were no effects of sex on the changes in VT$\dot{V}$O_2_ (*p* = 0.646) or the $\dot{V}$O_2_ peak (*p* = 0.064).

Supplement compliance was recorded with dosing logs and demonstrated a mean (±SD) compliance rate of 98.6% ± 2.6%. Additionally, the 3 × 2 mixed factorial ANOVAs resulted in no significant group × time interactions (F = 0.222–0.866, *p* = 0.430–0.802, $\eta_{p}^{2}$ = 0.013–0.047), main effects for group (F = 0.434–1.572, *p* = 0.222–0.652, $\eta_{p}^{2}$ = 0.024–0.082), or main effects for time (F = 0.027–0.956, *p* = 0.335–0.870, $\eta_{p}^{2}$ = 0.001–0.027) for the total kilocalories or macronutrients consumed. The reported average caloric intake per day (over 6 days) across all three groups was 1639 ± 775 kilocalories⋅d^−1^ and the total grams consumed per day for carbohydrates, protein, and fat were 186 ± 80 g⋅d^−1^, 79 ± 40 g⋅d^−1^, and 64 ± 38 g⋅d^−1^, respectively. Furthermore, there was no significant group × time interaction (F = 0.799, *p* = 0.457, $\eta_{p}^{2}$ = 0.037), main effect for group (F = 0.980, *p* = 0.384, $\eta_{p}^{2}$ = 0.045), or main effect for time (F = 2.458, *p* = 0.124, $\eta_{p}^{2}$ = 0.055) for body mass (Table 1).

### 3.2. Reliability

There were no significant mean differences between PRE- and POST-test for the VT$\dot{V}$O_2_ (*t* = 1.224, *p* = 0.244, *d* = 0.20) or $\dot{V}$O_2_ peak (t = −0.293, *p* = 0.775, *d* = 0.10) for the PLA group. The ICC values for the VT$\dot{V}$O_2_ and $\dot{V}$O_2_ peak were 0.959 and 0.971, respectively. The SEM and MD values for the VT$\dot{V}$O_2_ and $\dot{V}$O_2_ peak are presented in Table 2.

### 3.3. Fatigue Thresholds and Maximal Testing Parameters

**Figure 3:**
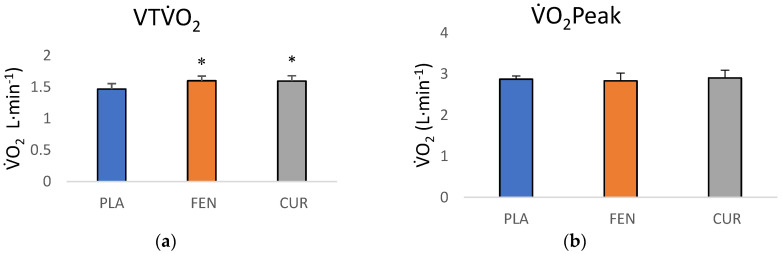
(**a**) Adjusted POST-test ventilatory threshold (VT) $\dot{V}$O_2_ (mean ± SD) values (covaried for PRE-test VT$\dot{V}$O_2_ scores) for placebo (PLA), fenugreek (FEN), and the CurQfen^®^ (CUR) groups. * Significantly (*p* < 0.05) greater than placebo; (**b**) adjusted POST-test $\dot{V}$O_2_ peak (mean ± SD) values (covaried for PRE-test $\dot{V}$O_2_ Peak scores) PLA, FEN, and the CUR groups.

The one-way ANCOVA for the VT$\dot{V}$O_2_ values indicated there were significant differences among the groups (F = 3.224, *p* = 0.050, $\eta_{p}^{2}$ = 0.136). The pairwise comparisons indicated a significant difference between the CUR and PLA groups (*p* = 0.039, *d =* 0.82) and between the FEN and PLA groups (*p*= 0.025, *d =* 0.85), but no differences between FEN and CUR groups (*p* = 0.943, *d* = 0.025). The adjusted VT$\dot{V}$O_2_ mean (±SD) for the PLA, FEN, and CUR were 1.465 ± 0.155 L·min^−1^ (95% CI = 1.378, 1.552 L·min^−1^), 1.597 ± 0.157 L·min^−1^ (95% CI = 1.522, 1.671 L·min^−1^), and 1.593 ± 0.157 L·min^−1^ (95% CI = 1.509, 1.677 L·min^−1^), respectively (Figure 3a). The one-way ANCOVA for $\dot{V}$O_2_ peak (F = 0.613, *p* = 0.547, $\eta_{p}^{2}$ = 0.029) indicated there were no significant differences among groups (Figure 3b). The adjusted $\dot{V}$O_2_ peak mean (±SD) for the PLA, FEN, and CUR were 2.872± 0.184 L·min^−1^ (95% CI = 2.758, 2.975 L·min^−1^), 2.831 ± 0.187 L·min^−1^ (95% CI = 2.743, 2.919 L·min^−1^), and 2.903 ± 0.183 L·min^−1^ (95% CI = 2.803, 3.003 L·min^−1^), respectively (Figure 3b). Additionally, the one-way ANCOVA for RER at the VT (F = 0.622, *p* = 0.542, $\eta_{p}^{2}$ = 0.029) indicated there were no significant differences among groups. The adjusted RER mean (± SD) for the PLA, FEN, and CUR were 0.970 ± 0.058 (95% CI = 0.938, 1.002), 0.948 ± 0.059 (95% CI = 0.920, 0.976), and 0.965 ± 0.060 (95% CI = 0.934, 0.997), respectively.

### 3.4. Individual Responses for Ventilatory Threshold ($VT\dot{V}$O_2_) and $\dot{V}$O_2_ Peak

**Figure 4:**
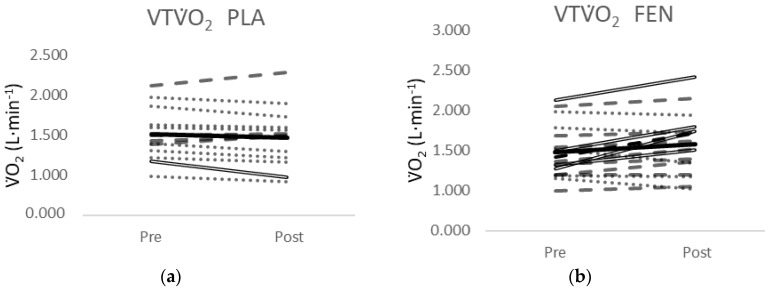

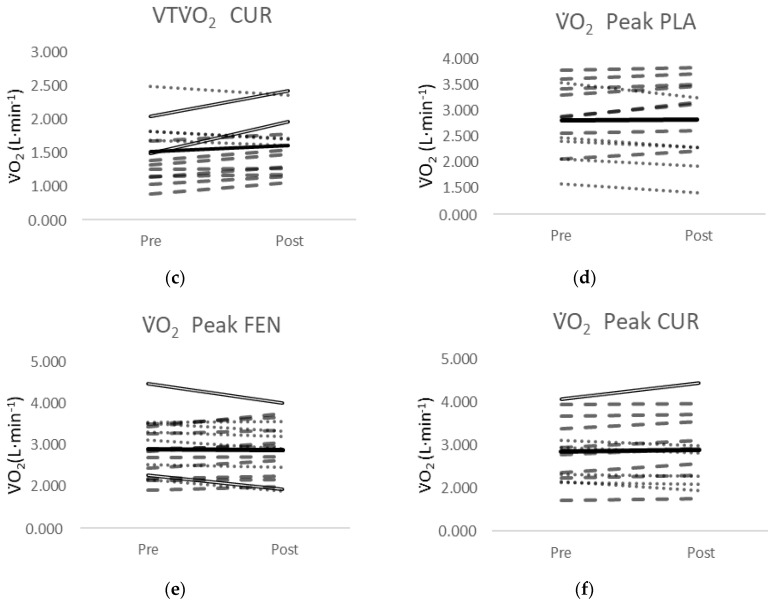
(**a**) Individual responses for the VT$\dot{V}$O_2_ from PRE- to POST-test for the placebo (PLA) supplement group; (**b**) individual responses for the VT$\dot{V}$O_2_ from PRE- to POST-test for the fenugreek (FEN) supplement group; (**c**) individual responses for the VT$\dot{V}$O_2_ from PRE- to POST-test for the CurQfen^®^ (CUR) supplement group; (**d**) individual responses for the $\dot{V}$O_2_Peak from PRE- to POST-test for the placebo (PLA) supplement group; (**e**) individual responses for the $\dot{V}$O_2_Peak from PRE- to POST-test for the fenugreek (FEN) supplement group; (**f**) individual responses for the $\dot{V}$O_2_Peak from PRE- to POST-test for the CurQfen^®^ (CUR) supplement group. Dashed lines indicate a positive slope and the dotted lines indicate a negative slope from PRE- to POST-test. Solid double lines indicate an increase or decrease greater than the minimal difference. The black line indicates the mean response.

One of the 13 participants in the PLA group showed a decrease greater than MD for the VT$\dot{V}$O_2_ (Figure 4a). Four of the 18 participants in the FEN group (Figure 4b) and two of the 14 participants from the CUR group (Figure 4c) showed an increase greater than MD for the VT$\dot{V}$O_2_. None of the 13 participants in the PLA group showed a change in $\dot{V}$O_2_ peak greater than the MD (Figure 4d). Two of the 18 participants in the FEN group (Figure 4e) showed a decrease greater than the MD and one of the 14 participants in the CUR Figure 4f) showed an increase greater than the MD.

## 4. Discussion

The purpose of this study was to examine the effects of a 28-day dosing period of curcumin and fenugreek soluble fiber on submaximal and maximal endurance performance. The primary findings were that the VT was greater for the CUR and FEN compared to the PLA at POST-test, but there were no differences in the $\dot{V}$O_2_ peak values among the groups. In this study, the VT increased 6.2% (increase = 0.094 L·min^−1^) and 6.7% (increase = 0.099 L·min^−1^) from PRE- to POST-test for the CUR and FEN, respectively, but was not improved for the PLA (−2.2%, decrease = 0.034 L·min^−1^). These mean responses, however, reflected increases above the MD for only 4 of the 18 participants in the FEN group and 2 of the 14 participants in the CUR group. To our knowledge, no previous studies have examined the effects of curcumin and fenugreek on submaximal fatigue thresholds; however, the relative changes (6.2–6.7%) in the VT in this study were consistent with the 4.1% to 5.4% increases previously reported for the gas exchange threshold (GET) after 28 days of arginine supplementation [34]. Interestingly, these increases in the GET were also not accompanied by changes in $\dot{V}$O_2_peak. Thus, the results of the present study showed increases one the submaximal fatigue threshold (VT) for both CUR and FEN, without changes in maximal endurance performance indices, that were consistent with previously reported [34] changes in a similar threshold after a nutritional intervention. Furthermore, the similar responses for CUR and FEN groups indicated that it is likely that the fenugreek soluble fiber was responsible for the observed effects.

### 4.1. Supplementation Effects on a Submaximal Endurance Performance Threshold

Although there is conflicting evidence regarding the true underlying mechanism(s) for the breakpoints in the $\dot{V}$_E_ versus $\dot{V}$O_2_ and $\dot{V}$CO_2_ versus $\dot{V}$O_2_ relationships that define the VT and GET, respectively, these thresholds have been demonstrated across multiple studies [22,25,35] and are likely related to the accumulation of metabolic byproducts (i.e., H^+^, inorganic phosphate, ammonia, and potassium) of muscular contractions. The VT and GET demarcate the moderate to heavy exercise intensity domains and reflect the point of increased reliance on anaerobic ATP production, as the aerobic system can no longer fully support the energy demands of the exercise intensity [36]. Previously, it was hypothesized that increases in the GET after arginine supplementation were related to the essential role of the amino acid in the synthesis of NO production and the subsequent vasodilatory response to enhance metabolic byproduct clearance [34,35]. One of curcumin’s purported physiological benefits is the upregulation of enzymes involved in NO production and enhanced acetylcholine-induced vasodilation [2,7]. Nitric oxide bolsters tissue respiration and endothelium-dependent vasodilation by relaxing smooth muscle cells in the vasculature [37,38]. In addition, curcumin supplementation has been shown to reduce the accumulation of metabolic byproducts (lactate and ammonia) of muscular contraction in rodents and humans [19,20,21]. It is possible the reduction in these metabolites after curcumin supplementation were a result of increased NO production and enhanced endothelium-dependent vasodilation [2]. In the current study, however, the VT for the CUR group was not increased above that of the FEN group alone. Thus, the changes in the VT in this study were likely not related to increased metabolic byproduct clearance from NO-induced vasodilation, but rather, were likely driven by the effects of fenugreek soluble fiber.

Fenugreek soluble fiber, also known as galactomannan, was added to curcumin (CurQfen^®^) to increase the bioavailability of the supplement [7]. Theoretically, galactomannans slow digestion, especially in the small intestine, resulting in a greater absorption of curcumin and greater plasma curcumin concentrations [8]. However, galactomannans from fenugreek have also been shown to have physiological effects after 28 days of supplementation. Two of the purported benefits of chronic galactomannan supplementation are an increased plasma FFA concentration in circulation and decreased plasma insulin levels [13,14]. It has been shown [17] that FFA oxidation rates are increased by greater concentrations of FFAs in circulation. During exercise in the moderate domain (i.e., below the VT), FFAs are the primary energy substrate for aerobic ATP production. Thus, greater plasma FFAs in circulation may increase the rate of FFA utilization, potentially delaying the reliance on anaerobic glycolytic metabolism and attenuating metabolic byproduct accumulation. Furthermore, the supplementation of galactomannans from fenugreek has been shown to increase plasma insulin sensitivity, decrease plasma insulin levels, and decrease blood glucose levels in mice models, and has been replicated in human models for both fasting and post-oral glucose tests [13]. Insulin suppresses lipolysis by directly inhibiting the transcription of lipase via the mTOR pathway [39,40]. Increased insulin sensitivity and subsequent decreases in insulin levels would, theoretically, increase lipolysis and favor fat mobilization. Indeed, previous investigators have reported a significant, positive relationship between insulin sensitivity and oxidative capacity [14,41]. Thus, it is possible the VT was improved in the FEN and CUR groups from increased FFA oxidation that delayed reliance on anaerobic glycolysis and attenuated the accumulation of metabolic byproducts. Future studies should further examine the effects of fenugreek, particularly the galactomannan component, on FFA concentrations and insulin sensitivity to determine its relationship with submaximal exercise performance indices.

### 4.2. Synergistic Effects of Curcumin and Galactomannan Soluble Fiber

It is likely the purported effects of fenugreek were responsible for the increases in VT for the CUR group (CurQfen^®^ = curcumin + fenugreek: 300 mg). Due to the poor bioavailability of curcumin, it is difficult to achieve plasma curcumin levels of physiological effect without a bioavailability booster such as fenugreek or piperine [7,8]. Therefore, we could not isolate the individual effects of curcumin in this study. Based on the purported effects of curcumin and fenugreek, it would seem logical that the combination of both would exhibit synergistic effects to improve performance. Unexpectedly, both the CUR and FEN group demonstrated a greater VT at POST-test compared to the placebo, but the VT was not different between the CUR and FEN. In this study, the 500 mg dose of CurQfen^®^ contained 190 mg of curcuminoids and 300 mg of fenugreek soluble fiber (75–80% galactomannans). It is possible at this relative dosage that any differences between the supplementation groups (CUR and FEN) were too small to detect. Therefore, the results of the present study indicated that fenugreek soluble fiber (galactomannan), and not curcumin, was responsible for the observed changes in the submaximal aerobic performance index (VT). Future studies should examine the effects of supplementation with various doses of curcuminoids, without additional fenugreek fiber, to determine if there are any differences between curcumin and fenugreek supplementation on the VT. In addition, future studies should examine the effects of supplementation of fenugreek fiber alone and curcumin in combination with other ingredients (to increase absorption), such as piperine, to determine if there are similar changes in submaximal endurance performance indices.

### 4.3. Supplementation Effects on Maximal Endurance Measurement ($\dot{V}$O_2_peak)

Curcumin and fenugreek soluble fiber supplementation had no effect on $\dot{V}$O_2_peak in this study. The VT and GET may be more sensitive to interventions affecting aerobic adaptations such as oxygen supply and substrate availability, while the $\dot{V}$O_2_peak may be more sensitive to changes affecting anaerobic metabolic system buffering capacities [22,25,42]. Thus, it is possible that NO-mediated vasodilation and increased FFA concentrations as a result of curcumin and fenugreek supplementation, respectively, were effective in improving aerobic metabolic efficiency and the VT, but did not alter the cellular and blood buffering capacities (e.g., carnosine and sodium bicarbonate, respectively) that would increase maximal endurance. Furthermore, the lack of change in $\dot{V}$O_2_peak after curcumin or fenugreek supplementation may also be related to the mechanisms of action of the supplements and the mode of testing. Specifically, the previous literature has demonstrated that increased local vasodilation did not equate to a higher local and systemic $\dot{V}$O_2_peak during maximal incremental studies [43]. Thus, the potential NO mediated vasodilation and increased metabolic byproduct clearance as a result of curcumin supplementation would likely not alter $\dot{V}$O_2_peak. In addition, curcumin supplementation has been reported to increase glycogen stores by 1.39- to 1.49- fold in mice [19]. Because our incremental test was designed to encourage failure and $\dot{V}$O_2_peak within 12 to 15 min, it is unlikely that the muscle or liver glycogen stores were depleted and, therefore, would not limit this parameter ($\dot{V}$O_2_peak). The primary action of galactomannans to slow digestion, increase insulin sensitivity, and decrease blood glucose to promote FFA oxidation appeared to be ineffective at altering measures of maximal performance after 28 days of supplementation in this study. These findings are supported by the previous literature that reported no effects on $\dot{V}$O_2_max after eight weeks of FEN supplementation [44]. Thus, in healthy, untrained participants, it seems that chronic, non-stimulant spice-related nutritional supplementation affects submaximal thresholds that demarcate the moderate to heavy domains but are not effective for higher thresholds or maximal performance indices ($\dot{V}$O_2_peak). Future studies should examine the effects of curcumin and/or fenugreek on 

$\dot{V}$O_2_peak at a submaximal intensity, such as the VT, to examine potential effects in order to improve the sustainability of aerobic exercise.

### 4.4. Individual Responses

Typically, overall conclusions regarding the effectiveness of an intervention are drawn from mean responses; however, the MD analyses in this study indicated there was a small percentage of participants that demonstrated a real change in the VT in the CUR and FEN supplementation groups. In this study, although there were significant effects of supplementation on the mean VT responses at POST-test for the FEN and CUR groups (Figure 3b,c) compared to PLA (Figure 3a), and the mean responses of the groups were similar (CUR = 6.2%, FEN = 6.7%), only four of the 18 participants (22%) exceeded the MD to be considered a real increase in the FEN group, and two of the 14 participants (14%) exceeded the MD in the CUR group. Conversely, no participants in the PLA group demonstrated an increase in the VT that exceeded the MD, but one of the 13 participants (7.7%) exceeded the MD to be considered a real decrease. The MD is defined as “the difference needed between separate measures on a subject for the difference in the measures to be considered real” [32] and speaks to the sensitivity of the test in distinguishing a “real” change from variation or error in measurement. A greater number of participants in the FEN group exceeded the MD compared to the CUR group, and 71.4% of the participants in the CUR-supplemented group demonstrated a positive slope coefficient from PRE- to POST-test, compared to 66.6% of the participants in the FEN group, while only 30.7% demonstrated a positive slope coefficient for the PLA group. The inherent limitation of simplifying results to the mean response is the assumption that all individuals have the same metabolic structure and capacities, where biological variability and biological noise such as circadian rhythm, nutritional intake, and motivation are not accounted for [45,46]. Thus, the mean responses for the CUR and FEN groups reflected a small percentage (14–22%) of participants that demonstrated a real change and a larger percentage (67–72%) of participants that demonstrated small increases that would not be considered real changes.

A further understanding of the underlying mechanisms related to the relatively small percentage of participants who demonstrated a real change would likely require the measurement of additional biomarkers. We did not measure any physiological markers outside of resting blood pressure, heart rate, and patient self-reported medical history to confirm that the participants were healthy and asymptomatic of any metabolic, cardiovascular, renal, or pulmonary diseases. However, baseline measurement of other markers such as arterial stiffness, lipid profiles, total cholesterol, fasting glucose level, and plasma insulin may have better informed the likelihood of demonstrating responses to an intervention. Based on previous evidence [2,5,13,14], it appears participants with above average arterial stiffness, hypertension, endothelial dysfunction, and insulin resistance may be more sensitive to the effects of curcumin and fenugreek soluble fiber interventions. It is possible that the participants who exceed the MD in this study might have had biological differences affecting sensitivity to the nutritional interventions. In addition, the responsiveness to an intervention is also likely related to an individual’s genotype. For example, genetic predisposition has been shown to influence differences in low and high responders regarding hypertrophic changes specific to resistance exercise [47]. Participants that were homozygous for a specific genotype or allele expression were observed to experience greater or lower degrees of hypertrophy [47]. These observations were centered on hypertrophy responsiveness; however, it is possible that genetic variances may make an individual more receptive to the effects of nutritional interventions and/or aerobic exercise interventions. Based on the current findings, we recommend that interventions be examined not only based on the mean response, but also on an individual-by-individual basis to provide further information on the sensitivity of the interventions (e.g., CurQfen^®^ and/or galactomannans supplementation) to affect performance outcomes. Furthermore, baseline measurement of arterial stiffness, lipid profiles, total cholesterol, fasting glucose levels, and plasma insulin, in addition to individual responses, should be considered to further examine the proportion of the population that may demonstrate a real change.

Factors related to study design might also help explain the individual variability in response to CurQfen^®^ and/or galactomannans supplementation. Specifically, the low percentage (four out of 18 = 22% and two out of 14 = 14%) of participants that exceeded the MD in this study may be related to the duration of the supplementation period, the relative dosage of supplementation, and/or the exclusion of an exercise intervention. It is possible that a longer supplementation period and/or a higher relative dosage are necessary for the effects of curcumin and/or galactomannan to fully manifest, as previous investigators have indicated a dose dependency [2,11,15,19]. Furthermore, this study did not include an exercise intervention or examine the benefits of curcumin on recovery or inflammation. Previous studies that have examined curcumin supplementation in conjunction with exercise have demonstrated a greater magnitude of change compared to PLA when the two interventions were combined [19,20,21]. These effects have been attributed to curcumin’s anti-inflammatory effects and enhanced recovery [2,19,20]. Thus, future studies should examine longer supplementation periods (>6 weeks) of curcumin and galactomannan at higher relative doses (>500 mg·day^−1^) in conjunction with an exercise training protocol to determine if the effects on the VT in this study for a few participants (14–22%) are extended to a larger portion of the sample.

### 4.5. Limitations

This study examined participants who were approximately 20–22 years of age. Therefore, these findings cannot be generalized to older individuals. Furthermore, we excluded participants who fell below fitness strata defined as “very poor” and “high fitness.” Thus, we cannot generalize our findings to individuals falling within those fitness categories, and further research is needed to ascertain the effectiveness of CUR and FEN in those individuals. Additionally, one of the primary limitations of the current study was the dependence on subject compliance. The participants were not confined to the laboratory throughout the supplementation and testing periods; therefore, sleep and dietary intake outside of the three days prior to PRE- and POST-testing were not accounted for. However, there were no differences in the macronutrient and total energy intakes from the self-reported three-day food logs at PRE- and POST-test. Measuring the physical activity and diet in the months prior to testing would have provided a baseline to determine if these habits changed during the intervention period. In addition, many of our participants were college-aged and it is possible the academic calendar and social stressors might have influenced their PRE- to POST-test responses. Furthermore, the laboratory availability for testing was limited and the time of day for PRE- to POST-test was kept consistent as much as possible but was not always identical. To control for these limitations as much as possible, we accounted for any prior supplementation through the health history review, as well as encouraged participants not to change exercise or dietary habits during enrollment.

## 5. Conclusions

The current findings indicated that fenugreek soluble fiber was responsible for the improvements in one submaximal threshold (VT), but did not alter $\dot{V}$O_2_peak after 28 days of CUR and FEN supplementation. The changes in the VT are most likely related to the increased FFA availability from fenugreek soluble fiber [2,13,14,15,17,19,20]. Previous investigators have indicated that curcumin had a small effect on $\dot{V}$O_2_peak in mice, and these effects may be amplified with the addition of an exercise intervention [19,20]. Thus, the lack of change in the $\dot{V}$O_2_peak in this study may be related to the inclusion of only a supplementation intervention without exercise. Potentially examining these same markers with an exercise intervention group might yield significant results that were not demonstrated with supplementation alone. The primary implications of the current study are that curcumin + fenugreek soluble fiber and fenugreek soluble fiber demonstrated equal effects on a submaximal exercise intensity. These findings demonstrate the potential for fenugreek soluble fiber to delay fatigue and improve aerobic performance in healthy, asymptomatic individuals. It is important for researchers and practitioners to note, however, that the mean responses for the CUR and FEN group reflected a change greater than the MD for 14% (two out of 14) and 22% (four out of 18) of participants, respectively. Based on these findings, we recommend that, in addition to mean responses, researchers and practitioners examine nutritional interventions on a participant-by-participant basis.

## Figures and Tables

**Table 1:** Demographic information (mean ± SD) for age, height and pre-test as well as body mass and BMI before (PRE) and after (POST) test.

	PLA		FEN		CUR	
	PRE	POST	PRE	POST	PRE	POST
Age (years)	20.5 ± 1.5		20.9 ± 1.4		22.1 ± 3.8	
Height (cm)	175.3 ± 7.6		173.5 ± 7.9		173.7 ± 9.1	
Body Mass (kg)	71.3 ± 11.0	71.5 ± 11.2	76.7 ± 13.0	76.7 ± 12.8	70.3 ± 16.0	70.8 ± 16.5
BMI (kg⋅m^−2^)	23.1 ± 2.6	23.1 ± 2.6	25.5 ± 3.9	25.5 ± 3.8	23.1 ± 3.0	23.3 ± 3.1

Placebo (PLA) (*n* = 13), Fenugreek (FEN) (*n* = 18), CurQfen^®^ (CUR) (*n* = 14), body mass index (BMI).

**Table 2:** Results of the reliability analyses for the placebo group using PRE-test and POST-test values for the ventilatory threshold (VT$\dot{V}$O_2_) and $\dot{V}$O_2_ peak in L⋅min^−1^.

Subject	$\mathrm{PRE}-\dot{V}O2_{\;}\mathrm{Peak}$	$\mathrm{POST}-\dot{V}O2_{\;}\mathrm{Peak}$	$\mathrm{PRE}-\mathrm{VT}\dot{V}O_{2}$	$\mathrm{POST}-\mathrm{VT}\dot{V}O_{2}$
1	3.782	3.826	1.500	1.520
2	2.876	3.164	2.118	2.287
3	2.413	2.282	1.310	1.220
4	3.537	3.245	1.860	1.730
5	2.065	1.921	1.170	0.980 *
6	3.423	3.509	1.971	1.890
7	2.472	2.289	1.400	1.300
8	3.614	3.715	1.430	1.484
9	2.890	3.127	1.390	1.500
10	1.578	1.403	0.990	0.925
11	2.565	2.609	1.626	1.592
12	2.057	2.219	1.224	1.162
13	3.308	3.464	1.599	1.561
**Mean ± SD**	**2.814 ± 0.691**	**2.829 ± 0.758**	**1.507 ± 0.325**	**1.473 ± 0.372**
**ICC**	**0.971**		**0.959**	
**SEM**	**0.119**		**0.066**	
**MD**	**0.330**		**0.183**	

Intraclass correlation coefficient (ICC); standard error of the measurement (SEM); minimal difference (MD) to be considered a real change. An increase or decrease from PRE-test to POST-test that exceeded the MD (*).Bolded: to differentiate the values from the individual responses above.
